# Cumulative meta-analysis and trial sequential analysis of correlation between hOGG1 Ser326Cys polymorphism and the risk of head and neck squamous cell carcinoma

**DOI:** 10.18632/oncotarget.24055

**Published:** 2018-01-06

**Authors:** Yan Yan, Ai-Ping Deng, Wen Chen, Yu-Hua Ming, Xian-Tao Zeng, Wei-Dong Leng

**Affiliations:** ^1^ Department of Stomatology, Taihe Hospital, Hubei University of Medicine, Shiyan 442000, China; ^2^ Department of Neurosurgery, Taihe Hospital, Hubei University of Medicine, Shiyan 442000, China; ^3^ Department of Radiology, Taihe Hospital, Hubei University of Medicine, Shiyan 442000, China

**Keywords:** human 8-oxoguanine glycosylase 1, hOGG1, polymorphism, head and neck squamous cell carcinoma, meta-analysis

## Abstract

**Background:**

The human 8-oxoguanine glycosylase 1 (*hOGG1*) Ser326Cys polymorphism has been involved in the risk of head and neck squamous cell carcinoma (HNSCC), but the results of published studies on this topic still inconsistent.

**Results:**

Finally 11 qualified publications with 13 independent case-control studies were yielded. Overall, we observed significant differences in CysCys vs. SerSer [odds ratio (OR) = 1.55, 95% confidence interval (95% CI) = 1.01–2.38] and CysCys vs. SerCys+SerSer (OR = 1.42, 95% CI = 1.005–1.99) genetic models. Sensitivity analyses showed the results were not robust, cumulative meta-analyses and trial sequential analysis indicated the results didn't not need more studies to identification. Subgroup analyses showed there was a significant association in Caucasian, laryngeal squamous cell carcinoma, studies agreement with Hardy-Weinberg equilibrium, and alcohol drinkers subgroups under the corresponding contrasts. In addition, the results of Egger’s test were contradictory.

**Materials and Methods:**

All eligible studies were searched from the online databases including PubMed, Web of Science, China Knowledge Resource Integrated Database, and Wanfang databases up to February 10, 2017. After study selection and data extraction, the meta-analysis was performed using STATA 12.0 software and TSA software version 0.9 Beta.

**Conclusions:**

Our meta-analysis results indicated that *hOGG1* Ser326Cys polymorphism may be associated with increased risk of HNSCC, especially in Caucasians, alcohol drinkers and the patients with laryngeal squamous cell carcinoma.

## INTRODUCTION

Head and neck squamous cell carcinoma (HNSCC), involving the oral cavity, pharynx, and larynx, constitutes 12% of all malignant neoplasms worldwide [1]. It is estimated that a total of 400,000 cases of the oral cavity and pharynx cancers and 160,000 cases of laryngeal cancer have been diagnosed and 300,000 people die of these diseases per year [2]. Hence, investigating and preventing the risk factors of HNSCC is a necessary and significant research field. In the past decades, many epidemiological researches have suggested that active and passive smoking, alcohol consumption, genetic factors, viral infection, gender, tooth loss, periodontal disease, and occupational exposure are the significant risk factors for HNSCC [3–12]. Of them, tobacco smoking and alcohol are supposed to the most important risk factors [3, 5]. However, such markers could not comprehensively explain the etiology of HNSCC [13]. That indicates that the individual susceptibility may play a certain role in the carcinogenesis of HNSCC.

With the rapid development of molecular epidemiology, some meta-analyses have provided increasing evidence to support the hypothesis that some genetic polymorphisms play a significant role in determining individual susceptibility to HNSCC [14–19]. DNA damage is considered as a critical factor to carcinogenesis. DNA repair mechanisms play an important role in the integrity and stability of the genome. Base excision repair (BER), which is an important DNA repair pathway, plays a vital role in the repair of mutations generated by reactive oxygen species (ROS). The 8-hydroxy-2 deoxyguanine (8-OH-dG) is one of the most abundant oxidative products of high mutagenesis among numerous factors of oxidative DNA damage, because it has the tendency to mispair with adenine during DNA replication and finally result in GC to TA mutation [20]. The human oxoguanine glycosylase 1 (*hOGG1*) is a DNA glycosylase or AP lyase, which has been investigated to play a vital role in preventing carcinogenesis by repairing oxidative damage to DNA [21]. The glycosylase or AP lyase could efficiently catalyze and remove 8-OH-dG adducts produced by reactive free radicals, which is a major form of DNA damage. It has been hypothesized that the polymorphism in *hOGG1* gene may affect the risk of developing HNSCC because of the critical roles in stabilizing genome integrity. The *hOGG1* gene has a G1245C polymorphism in exon 7 making the codon 326 coding Ser or Cys (rs1052133) [22], and Cys326 has lower ability to prevent mutagenesis by 8-OH-dG than Ser326 in human cells *in vivo* [21]. Many studies have suggested that this mutation may be associated with increased risk of several cancers [23]. Several of them have focused on the association of *hOGG1* Ser326Cys polymorphism with head and neck cancer risk. However, we observe that the results of the association between *hO*GG1 Ser326Cys polymorphism and HNSCC susceptibility remain controversial.

In 2011, Wei et al performed a meta-analysis based on 6 case-control studies indicating that *hOGG1* Ser326Cys polymorphism was significantly associated with HNSCC risk only under CysCys vs. SerSer model [24]. However, another meta-analysis of 6 case-control studies by Wang et al in 2012 [23] showed that there was a significant association under all five genetic models. Obviously, the results of these meta-analyses were inconsistent (Supplementary Table 1), which might be attributed to the small effect of the Ser326Cys variation on HNSCC risk or the relatively low statistical power of published papers. At present, eleven eligible articles [25–35] on this issue have been published. Hence, in order to more systematically investigate the association between *hOGG1* Ser326Cys polymorphism and risk of HNSCC, we included these eleven articles [25–35] and performed present updated meta-analysis through combing the different studies which a quantitative approach. For inspecting whether sample size influenced the overall results and judging whether more relevant studies would be worthwhile, we conducted not only cumulative analysis by cumulating the single study according to the publication year [36, 37] but also trial sequential analysis (TSA) to explore whether further studies are needed or not [38–40]. Additionally, we also preformed subgroup analyses according to ethnicity, tumor site, source of control, smoking, alcohol patients, genotyping method, and controls agreement with Hardy-Weinberg equilibrium (HWE), respectively.

## RESULTS

### Study identification and characteristics

**Figure 1:**
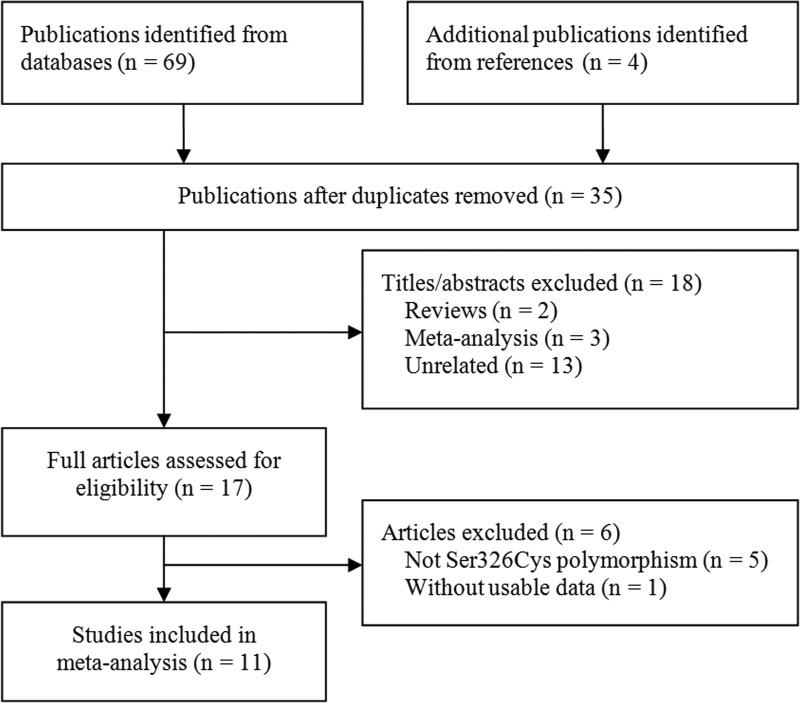
Flowchart of study section in the meta-analysis

We initially yielded 73 papers and finally 11 publications with 13 case-control studies [25–35] were included in the meta-analysis, involving a total of 3,875 patients and 4,696 healthy controls. Figure 1 presents the selection process.

Of them, the study by Hall et al [29] focused on many different tumor sites and it was considered as three independent trials. The published language of one study was in Chinese [30], and others were in English [25–29, 31–35]. The sample size of cases varied greatly, ranging from 29 to 706, and the number of controls ranged from 30 to 1196. Four studies involving six trials did not satisfy the HWE for control group [29, 33–35]. The main characteristics and quality of the included studies are shown in Table 1.

**Table 1:** Characteristics of all included studies in the meta-analysis

References	Country (Ethnicity)	Tumor site	Cases/ Controls				Source of control	Genotyping method	HWE
			N.	SerSer	CysSer	CysCys			
Elahi 2002	USA (Caucasian)	Oropharynx	169/338	104/249	54/76	9/6	PB	PCR-RFLP	Yes
Cho 2003	China (Asian)	Nasopharynx	334/283	36/46	175/129	122/108	PB	PCR-RFLP	Yes
Zhang 2004	USA (Caucasian)	HNSCC	706/1196	447/739	220/388	39/69	HB	PCR-RFLP	Yes
Gorgens 2007	Germany (Caucasian)	HNSCC	29/30	19/19	8/10	2/1	PB	PCR	Yes
Hall 1 2007	EE (Caucasian)	Oral cavity	160/754	98/485	52/253	1016	HB	Taqman	No
Hall 2 2007	EE (Caucasian)	Pharynx	107/754	65/485	35/23	7/16	HB	Taqman	No
Hall 3 2007	EE (Caucasian)	Larynx	312/754	206/485	98/23	8/16	HB	Taqman	No
Yang 2008	China (Asian)	Larynx	72/72	34/50	34/22	4/0	HB	PCR-RFLP	Yes
Pawlowska 2009	Poland (Caucasian)	Larynx	253/253	141/166	91/77	21/10	HB	PCR-RFLP	Yes
Laantri 2011	African (North African)	Nasopharynx	598/545	289/274	202/193	50/39	PB	TaqMan	Yes
Mitra 2011	India (Caucasian)	HNSCC	250/325	110/105	118/171	7/26	PB	PCR-RFLP	No
Sliwinski 2011	Poland (Caucasian)	Larynx	265/280	109/160	128/111	28/9	PB	PCR-RFLP	No
Tsai 2012	China (Asian)	Oral cavity	620/620	138/104	252/251	230/265	HB	PCR-RFLP	No

Notes: EE, Eastern European (Romania, Poland, Russia, Slovakia, and Czech Republic); African, Morocco, Algeria and Tunisia; HNSCC, head and neck squamous cell carcinoma; N., sample size; HWE, Hardy-Weinberg equilibrium.

### Meta-analysis

**Figure 2:**
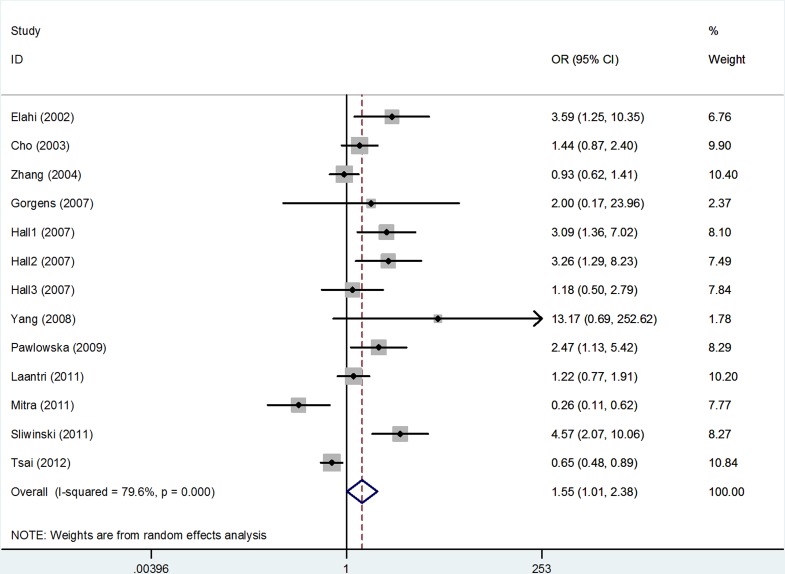
Forest plot of overall population based on CysCys vs. SerSer genetic model

**Figure 3:**
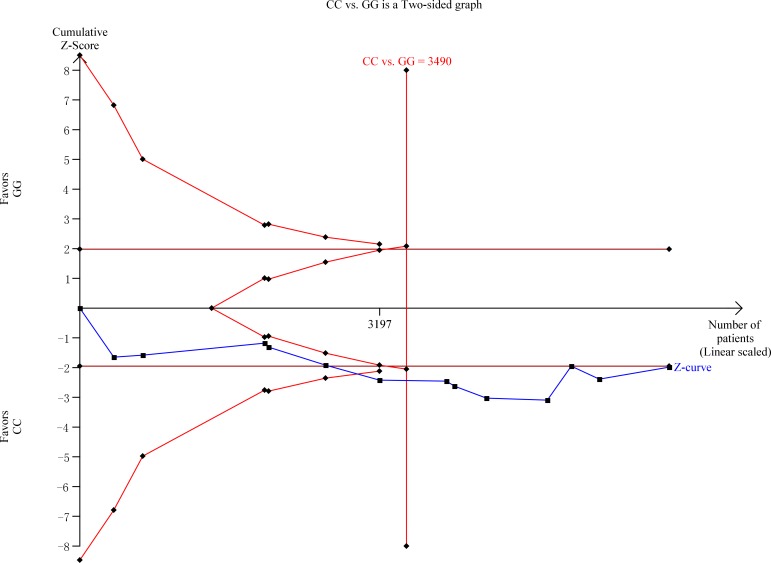
Trial sequential analysis of CysCys vs. SerSer genetic model CC, CysCys; GG, SerSer; a low risk diversity adjusted information size of 3490 patients was calculated using α = 0.05 (two sided), β = 0.20 (power 80%); TSA adjusted 95% confidence interval for a odds ratio of 1.55 is 1.01 to 2.38 based on random-effects model. TSA illustrated that the cumulative z curve crossed the conventional boundary and the trial sequential monitoring boundary for increased risk, and that the required information size was achieved, showing that currently cumulative evidence might be true positive

The estimation of the association between *hOGG1* Ser326Cys polymorphism and HNSCC risk is presented in Table 2. In consequence of the high degree of heterogeneity under all the genetic models, random-effects model was applied. Overall, the pooled analysis showed that a statistical significance under Cys/Cys vs. Ser/Ser [odds ratio (OR) = 1.55, 95%confidence interval (95% CI) = 1.01–2.38, *I*^2^ = 79.6%, Figure 2] and CysCys vs. (CysSer + SerSer) (OR = 1.42, 95% CI = 1.005–1.99, *I*^2^ = 74.3%) genetic models, but nonsignificant association under the other three genetic models [Cys vs. Ser: OR = 1.16, 95% CI = 0.98–1.37, *I*^2^ = 81.5%; CysSer vs. SerSer: OR = 1.11, 95% CI = 0.93–1.33, *I*^2^ = 67.2%; (CysCys+ CysSer) vs. SerSer: OR = 1.16, 95% CI = 0.95–1.42, *I*^2^ = 77.2%]. TSA showed that the overall results might be conclusive, as their cumulative z-curves crossed both the conventional boundary and the trial sequential monitoring boundary providing firm evidence (Figure 3).

**Table 2:** The results of overall and subgroup analysis of the all genetic models

	N.	Cys vs. Ser		CysCys vs. SerSer		CysSer vs. SerSer		(CysCys+ CysSer) vs. SerSer		CysCys vs. (CysSer + SerSer)	
		OR (95% CI)	*I*^2^ (%)	OR (95%CI)	*I*^2^ (%)	OR (95% CI)	*I*^2^ (%)	OR (95% CI)	*I*^2^ (%)	OR (95% CI)	*I*^2^ (%)
Overall	13	1.16 (0.98–1.37)	81.5	1.55 (1.01–2.38)	79.6	1.11 (0.93–1.33)	67.2	1.16 (0.95–1.42)	77.2	1.42 (1.005–1.99)	74.3
Ethnicity											
Caucasian	9	1.19 (0.95–1.49)	82.0	1.79 (0.97–3.30)	79.3	1.09 (0.88–1.34)	65.7	1.15 (0.90–1.47)	77.2	1.73 (1.01–2.97)	74.1
Asian	3	1.14 (0.75–1.75)	86.5	1.15 (0.49–2.71)	80.5	1.38 (0.68–2.80)	84.7	1.36 (0.63–2.93)	88.2	0.87 (0.65–1.17)	40.0
North African	1	1.06 (0.87–1.28)	NA	1.22 (0.77–1.91)	NA	0.99 (0.77–1.28)	NA	1.03 (0.81–1.31)	NA	1.22 (0.79–1.89)	NA
Tumor site											
Larynx	4	1.49 (1.04–2.13)	80.6	2.65 (1.27–5.54)	53.1	1.40 (0.97–1.33)	72.0	1.53 (1.01–2.33)	79.5	2.29 (1.29–4.08)	30.3
Oral cavity	2	0.98 (0.63–1.52)	85.8	1.36 (0.30–6.21)	91.7	0.86 (0.65–1.15)	31.2	0.89 (0.55–1.42)	77.3	1.47 (0.39–5.55)	90.1
Nasopharynx	2	1.07 (0.92–1.24)	0	1.31 (0.94–1.84)	0	1.26 (0.73–2.16)	74.2	1.22 (0.80–1.87)	62.8	1.03 (0.79–1.34)	0
Source of control											
Population-based	6	1.17 (0.86–1.60)	85.4	1.50 (0.72–3.12)	81.2	1.21 (0.85–1.72)	77.0	1.24 (0.84–1.83)	83.0	1.30 (0.72–2.36)	77.1
Hospital-based	7	1.14 (0.92–1.40)	78.3	1.60 (0.91–2.82)	79.4	1.02 (0.85–1.23)	50.9	1.09 (0.87–1.36)	70.0	1.56 (0.96–2.55)	75.4
Smokers	4	1.40 (0.78–2.50)	90.3	1.73 (0.54–5.56)	84.2	1.40 (0.78–2.51)	80.4	1.34 (0.69–2.62)	86.9	1.64 (0.67–3.98)	75.0
Alcohol drinkers	2	1.62 (1.23–2.13)	0	2.54 (0.72–8.95)	58.5	1.67 (1.19–2.35)	0	1.75 (1.26–2.42)	0	2.15 (0.58–8.02)	62.7
HWE (*P* > 0.05)	7	1.26 (1.03–1.55)	71.8	1.53 (1.04–2.24)	47.0	1.29 (1.00–1.65)	63.2	1.35 (1.04–1.76)	70.0	1.28 (0.92–1.78)	43.9
PCR-RFLP	8	1.20 (0.93–1.56)	88.7	1.42 (0.79–2.56)	84.9	1.23 (0.92–1.63)	80.0	1.26 (0.91–1.75)	86.3	1.22 (0.80–1.86)	77.8

Notes: OR, odd ratio; CI, confidence interval; N., number of case-control studies; HWE, Hardy-Weinberg equilibrium; NA, not available.

**Figure 4:**
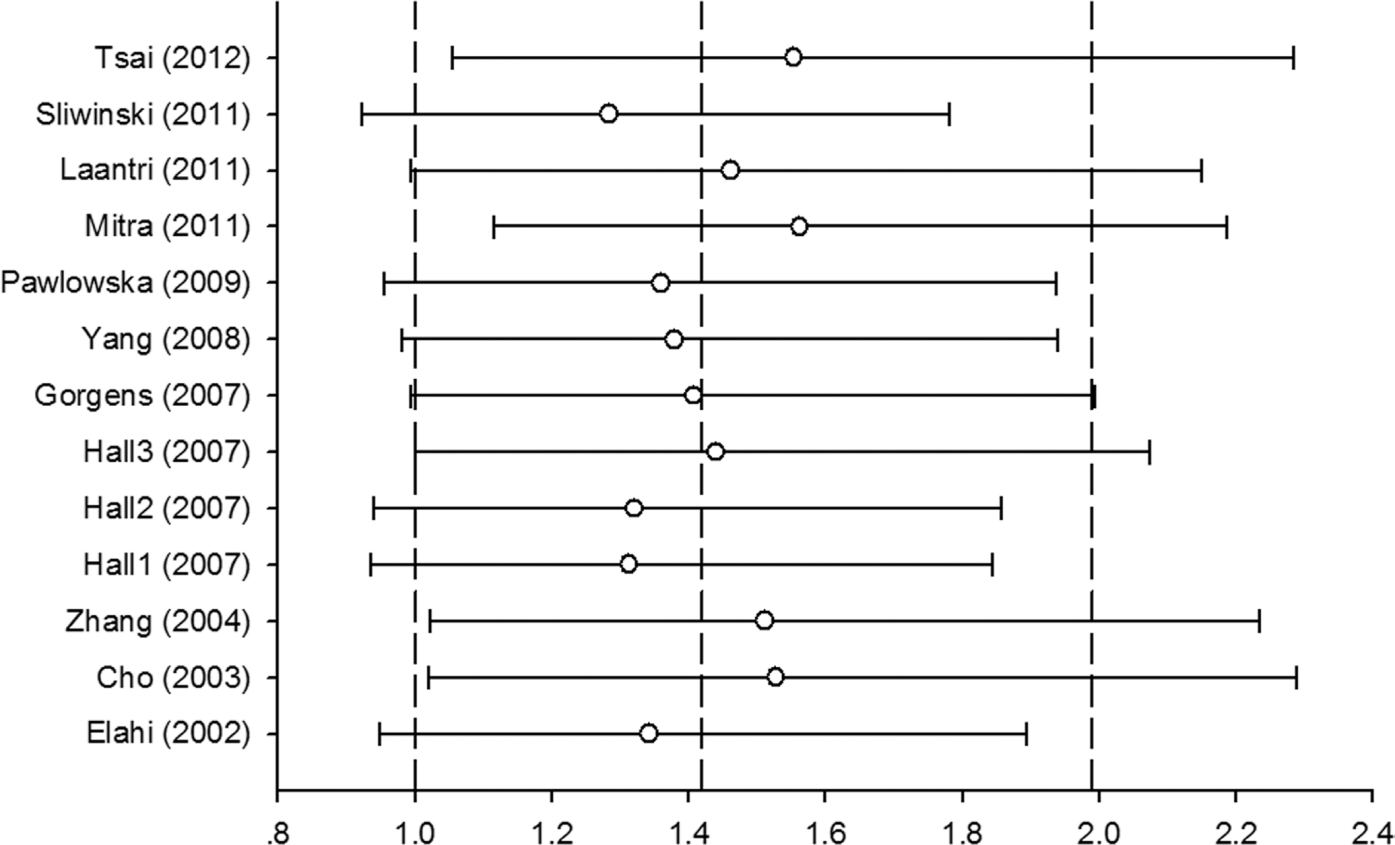
Forest plot of sensitivity analysis based on CysCys vs. SerSer model by omitting each study in turn

**Figure 5:**
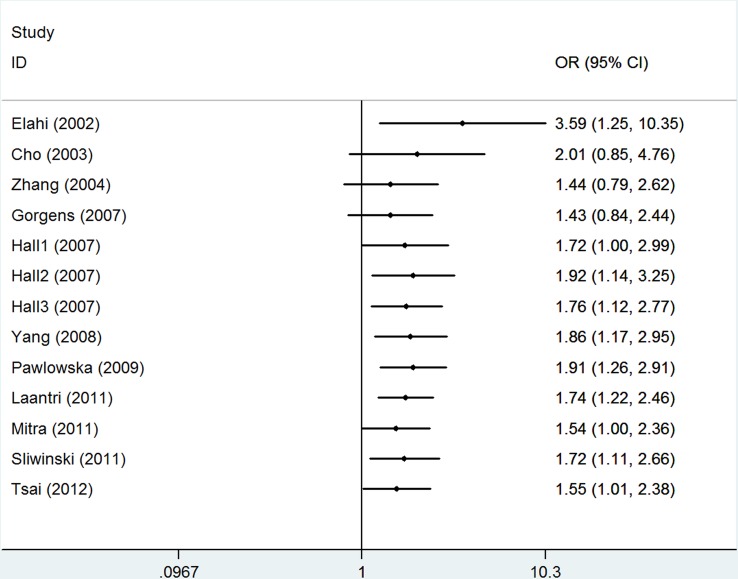
Forest plot of cumulative meta-analysis based CysCys vs. SerSer model by accumulating each study according to the publication year

We performed sensitivity analysis for testing the robustness of the pooled estimations, and the results showed that our findings were not robust under all the contrasts (Figure 4). The cumulative meta-analysis demonstrated the overall results from significant to nonsignificant to significant; the trend was not stable when the studies were accumulated (Figure 5).

To evaluate whether there was a different genotype effect in predefined subgroups of studies, we conducted stratified analyses in relation to ethnicity, tumor site, source of control, smoking, alcohol drinking, controls agreement with HWE, and genotyping method. Of them, we observed increased risks of Ser326Cys polymorphism for Caucasians, laryngeal squamous cell carcinoma (LSCC), alcohol drinkers and controls agreement with HWE under corresponding models. We did not find any significant association in other subgroups by control source, smokers, and genotyping method. Table 2 presented the results of subgroup analyses.

### Publication bias

**Figure 6:**
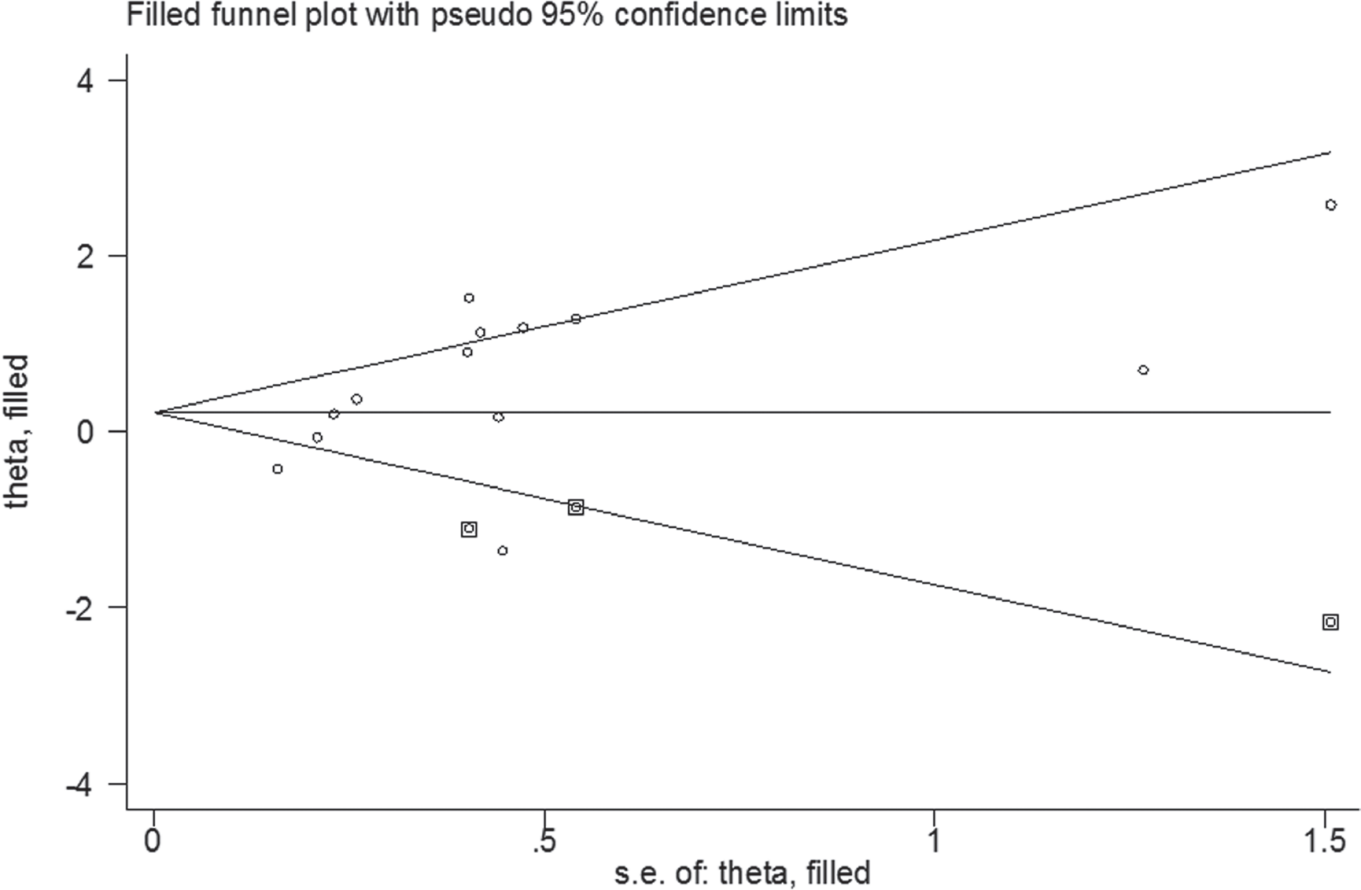
Funnel plots based on the trim and fill method of CysCys vs. SerSer model

Funnel plots based on the trim and fill method indicated obvious publication bias (Figure 6). Egger’s test also provided that funnel plots were asymmetry in the Cys vs. Ser (*p* = 0.048), CysCys vs. SerSer (*p* = 0.04), and CysCys vs. (SerCys+SerSer) (*p* = 0.02) genetic models; however, the publication bias was not detected in the CysSer vs. SerSer (*p* = 0.15) and (CysCys+ CysSer) vs. SerSer (*p* = 0.14) genetic models.

## DISCUSSION

The present meta-analysis including 3,875 HNSCC cases and 4,696 controls indicated that *hOGG1* Ser326Cys polymorphism might be associated with increased risk of HNSCC, and the TSA results also provided firm evidence. However, the results changed into nonsignificant after adjusting the publication bias by trim and fill method; sensitivity analysis and cumulative meta-analysis suggested the pooled ORs and corresponding 95%CIs were not robust. Minelli et al. [41] thought that papers that appear in which the controls deviated from HWE should not be excluded unless there were other factors affecting the quality of the study; Hence, we performed a subgroup analysis according to the controls agreement with HWE. The results reached statistical significance in allele model, co-dominant model, and dominant model. Based on stratified analysis by tumor site, we observed that the *hOGG1* Ser326Cys polymorphism was significantly associated with LSCC. However, only four studies focused on LSCC, that might reduce the reliability of the results. As we know, smoking and alcohol consumption have been identified as major risks for HNSCC [3, 5], hence, we preformed subgroup analyses according to smoking and alcohol drinking. There were four and two studies involving *hOGG1* Ser326Cys polymorphism and HNSCC in smokers and alcohol drinkers, respectively. Interestingly, we found no significant association between *hOGG1* Ser326Cys polymorphism and HNSCC in smokers but statistically significant relationship in alcohol drinkers. By carefully reading relevant papers, we found that the smokers were not stratified by smoking magnitude; nevertheless, it’s known that the power of influence of heavy smoking must be different from that of occasionally smoking. These results suggest that when designing a case-control study of genetic polymorphism and the risk of cancer relating to other factors, such as smoking- or alcohol-related cancer, researchers should stratify the smokers and drinkers based on magnitude, for giving a more reliable conclusion.

Comparing with the previous meta-analyses [23, 24], our meta-analysis has four advantages. First, the number of included studies and total sample sizes were larger. The number of included studies was six for both previous meta-analyses [23, 24], and the relevant results were contradictory. Of them, Wang et al [23] conducted a subgroup analysis focused on HNSCC containing six studies, which showed that *hO*GG1 Ser326Cys polymorphism increased HNSCC risk under all comparisons. The meta-analysis from Wei et al [24], included six studies and indicated a significant association between *hOGG1* Ser326Cys polymorphism and HNSCC under CysCys vs. SerSer model. Our meta-analysis incorporated 13 studies, which showed that *hOGG1* Ser326Cys polymorphism was associated with the elevated risk of HNSCC under CysCys vs. SerSer and CysCys vs. (CysSer + SerSer) contrasts. Moreover, we extracted the data of oral cavity, larynx, and nasopharynx cancers as well as the data of smokers and alcohol drinkers to performe relevant subgroup analyses. Relatively speaking, our paper was more scientific than previous studies on this topic, because it was the first one containing subgroup analyses based on the tumor sites, smokers, and alcohol drinkers. Third, we used cumulative meta-analysis and TSA to explore the results and found the relevant original studies didn’t need to be conducted continuously. Fourth, we assessed the methodological quality of included studies.

Meta-analysis is a retrospective analysis [42]; therefore, some limitations should be taken into consideration. First, the studies included in the present meta-analysis were medium-sized case-control studies, with little power to detect the final aggregated ORs. The results of cumulative analysis also provided that the sample size was not enough. Second, four included studies in our meta-analysis were not in HWE and a significant association was obtained under three genetic models based on HWE conformed studies. This indicated that the overall results might be not reliable. Third, large publication bias existed in this meta-analysis, which might affect the precision of our estimates. Furthermore, sensitivity analysis also showed the overall results were not robust. Fourth, the heterogeneity was moderate to high degree and it was failed to be explained by subgroup analyses. This might be induced by other internal validity, so we could not ignore the influence of heterogeneity. Fifth, although the result of methodological assessment of included studies got high scores, none of them reached the top score, indicating that the reliable of our results was influenced. Finally, like other genetic association meta-analyses [17, 23, 43], this meta-analysis was based on unadjusted data due to lack of detailed information stratified by main confounding variables from primary studies. Hence, gene-gene and gene-environment interactions results remain unclear.

In summary, our meta-analysis suggests that *hOGG1* Ser326Cys polymorphism may contribute to the occurrence of HNSCC, especially in Caucasians, alcohol drinkers and the patients with LSCC. However, due to the aforementioned limitations, the results of current meta-analysis should be treated with caution.

## MATERIALS AND METHODS

This meta-analysis was reported in accordance with the Meta-Analysis of Observational Studies in Epidemiology (MOOSE) guideline [44].

### Eligible criteria

Each study included in the present meta-analysis was required to meet all the following criteria: (i) the study focused on the association between *hOGG1* Ser326Cys polymorphism and risk of HNSCC; (ii) the study design was a cohort or case-control study; and (iii) the data of genotype distributions in case and control groups were available. The language was restricted to English and Chinese.

### Search strategy

A comprehensive electronic search was performed in PubMed, Web of Science, CNKI (Chinese National Knowledge Infrastructure) and Wanfang database up to February 10, 2017. The following search terms were used: (“human oxoguanine glycosylase 1” OR “hOGG1” OR “human 8-oxoguanine glycosylase 1”) AND (“head and neck” OR “oral” OR “pharyngeal” OR “oropharyngeal” OR “laryngeal” OR “tongue” OR “mouth”) AND (“cancer” OR “carcinoma”) AND (“polymorphism” OR “variant”). We also conducted a manual search of references cited in include studies and previously meta-analyses [24, 45] for additional pertinent studies.

### Data extraction

Two authors independently selected included studies and extracted essential data was extracted from all eligible studies, which contained surname of the first author, year of publication, country of study performed, ethnicity of descent, source control, HWE of control, tumor site, sample size, number of genotype distribution, genotyping method, and relevant methodological information. A cross-check over these data was performed by these two authors; any disagreements would be settled through discussion until reaching a final consensus.

### Data analysis

All the data analyses were applied using the STATA 12.0 software. HWE test of control group was performed by chi-square test. Heterogeneity among the included studies was tested by the *I*^2^ value [46]. If *I^2^* < 25% indicating the homogeneity was good, the fixed effect model was used; otherwise, random-effects model was applied. The OR and its 95% CI were calculated to estimate the overall and subgroup results. Subgroup analysis was used to explore and explain the diversity of the results in different researches using the following factor as stratifying variables: ethnicity, tumor site, source of control, smokers, alcohol drinkers, agreement with HWE in control group, and genotyping method. TSA was performed using diversity-adjusted information size based on α = 0.05, β = 0.20 (power at 80%), information size with estimate, and case and control event proportion calculated from meta-analysis, through TSA software version 0.9 Beta [38–40].

The sensitivity analysis was conducted by deleting every included study each time to investigate the influence of overall results [43]. The cumulative analysis was performed to assess the change trend with the sample size accumulated according to the publication year [36]. The funnel plot and Egger’s test were used to evaluate the underlying publication bias, and trim and fill method was applied if significant publication bias existed in the meta-analysis.

## SUPPLEMENTARY MATERIALS TABLE


