# Supplementary material for: Cumulative meta-analysis and trial sequential analysis of correlation between hOGG1 Ser326Cys polymorphism and the risk of head and neck squamous cell carcinoma
Source: Oncotarget. 2018 Jan 6;9(16):13077–87. doi: 10.18632/oncotarget.24055 (PMC5849197; doi:10.18632/oncotarget.24055)
Supplement: Supplementary file 1 [file oncotarget-09-13077-s001.pdf]

Cumulative meta-analysis and trial sequential analysis of correlation between hOGG1 Ser326Cys polymorphism and the risk of head and neck squamous cell carcinoma

SUPPLEMENTARY MATERIALS

Supplementary Table 1: Characteristics of published 2 meta-analyses

References	No. of studies	Included studies	Journal of publication	OR (95% CI)	Authors' conclusion
Wei 2011	4	Elahi 2002; Cho 2003; Zhang 2004; Gorgens 2007; Laantri 2011; Sliwinski 2011	PLoS ONE	Cys/Cys vs. Ser/Ser: 1.71 (1.05–2.78);<br>Cys/Ser vs. Ser/Ser: 1.27 (0.97–1.66);<br>Cys/Cys + Cys/Ser vs. Ser/Ser: 1.32 (0.99–1.77);<br>Cys/Cys vs. Cys/Ser+Ser/Ser: 1.41 (0.93–2.12)	No association
Wang 2012	6	Elahi 2002; Cho 2003; Zhang 2004; Yang 2008; Pawlowska 2009; Sliwinski 2010	Mutagenesis	Cys allele versus Ser allele: 1.43 (1.09–1.88)<br>CysCys versus SerSer: 2.19 (1.20–4.01)<br>SerCys versus SerSer: 1.48 (1.11–1.99)<br>SerCys/CysCys versus SerSer: 1.58 (1.14–2.19)<br>CysCys versus SerSer/SerCys: 1.73 (1.02–2.94)	Had a significantly susceptibility
